# On-target off-tumor toxicity; when enhancing an NKG2D-based CAR in vitro led to severe toxicities *in vivo*

**DOI:** 10.1186/2051-1426-2-S3-P15

**Published:** 2014-11-06

**Authors:** Heather VanSeggelen, Joanne A Hammill, Daniela GM Tantalo, Carole Evelegh, Galina F Denisova, Brian Rabinovich, Jacek M Kwiecien, Jonathan L Bramson

**Affiliations:** 1 McMaster University, Hamilton, Ontario, Canada; 2 M.D. Anderson Cancer Center, Houston, TX, USA

## 

Engineering T cells with chimeric antigen receptors (CARs) has emerged as a promising approach to adoptive T cell therapy for cancer. We have been studying CARs that employ the ligand-binding domain of the NKG2D receptor to target tumors. NKG2D ligand expression is increased on the surface of stressed cells, like tumor cells, making this family of ligands a target of interest for cancer immunotherapies. Two CARs were constructed: 1) a fusion of the full-length NKG2D receptor and CD3z (NKG2Dζ) and 2) the extracellular domain of NKG2D fused to a second-generation CAR scaffold composed of transmembrane and intracellular domains from CD28 and the signaling domain of CD3z (NKG2D28ζ). Since surface expression of NKG2D is limited by the presence of DAP10, we also created a vector where DAP10 was co-expressed with NKG2Dζ (NKG2Dζ10). Indeed, NKG2Dζ10-CAR-T cells displayed a greater than 10-fold increase in CAR expression compared to NKG2Dζ; NKG2D28ζ showed an intermediate level of expression. T cells expressing any of the NKG2D CARs produced IFNg and TNFa in response to NKG2D ligand stimulation and efficiently killed tumor targets *in vitro*. However, following infusion into syngeneic hosts, we observed significant toxicity *in vivo *with these CAR constructs. Signs of toxicity, including poor body condition, hunched posture, labored breathing, and decreased core body temperature were observed in tumor-bearing and tumor-free mice treated with NKG2D-based CAR-T cells as compared to control mice. The severity of NKG2D CAR-T cell toxicity varied, with NKG2Dζ10 being severely toxic, NKG2D28ζ showing intermediate toxicity, and NKG2Dζ being tolerable. Clinical symptoms of toxicity and mortality rates were exacerbated when mice received chemotherapy prior to adoptive transfer of T cells expressing *any *of the NKG2D CARs. These observations were consistent between BALB/c and C57BL/6 hosts. Further characterization revealed that the toxicity coincided with a systemic cytokine storm and lethal levels of inflammation within the lungs. These data warn that extreme caution should be taken when using NKG2D ligands for targeted immunotherapy and demonstrate that enhancing T cell expression of strongly activating CARs can be detrimental *in vivo *when the CAR target is not uniquely expressed on the tumor.

*This research was supported by the CIHR and the TFRI*.

